# Hepatoprotective and Antioxidative Activities of *Cornus officinalis* against Acetaminophen-Induced Hepatotoxicity in Mice

**DOI:** 10.1155/2012/804924

**Published:** 2011-08-17

**Authors:** Nam-Hun Lee, Chang-Seob Seo, Ho-young Lee, Da-Young Jung, Jun-Kyung Lee, Jin-Ah Lee, Kye Yong Song, Hyeun-kyoo Shin, Mee-Young Lee, Young Bae Seo, Hokyoung Kim, Hyekyung Ha

**Affiliations:** ^1^Herbal Medicine EBM Research Center, Korea Institute of Oriental Medicine, 483, Exporo, Yuseong-gu, Daejeon 305–811, Republic of Korea; ^2^College of Medicine, Chung-Ang University, Seoul 156–756, Republic of Korea; ^3^College of Oriental Medicine, Daejeon University, Daejeon 300–716, Republic of Korea; ^4^Herbal Resources Research Center, Korea Institute of Oriental Medicine, Daejeon 305–811, Republic of Korea

## Abstract

The fruit of *Cornus officinalis *Sieb. et Zucc. is commonly prescribed in Asian countries as a tonic formula. In this study, the hepatoprotective effect of ethanolic extracts of the fruit of *C. officinalis* (ECO) was investigated in a mouse model of acetaminophen- (APAP-) induced liver injury. Pretreatment of mice with ECO (100, 250, and 500 mg/kg for 7 days) significantly prevented the APAP (200 mg/kg) induced hepatic damage as indicated by the serum marker enzymes (AST, ALT, and LDH). Parallel to these changes, ECO treatment also prevented APAP-induced oxidative stress in the mice liver by inhibiting lipid peroxidation (MDA) and restoring the levels of antioxidant enzymes (SOD, CAT, and HO-1) and glutathione. Liver injury and collagen accumulation were assessed using histological studies by hematoxylin and eosin staining. Our results indicate that ECO can prevent hepatic injuries associated with APAP-induced hepatotoxicity by preventing or alleviating oxidative stress.

## 1. Introduction

The fruit of *Cornus officinalis *Sieb. et Zucc. (Corni Fructus) is commonly prescribed in traditional medicine as a tonic formula as well as a treatment for a wide range of medical conditions [1]. The components of this plant include iridoid total glycosides (such as morroniside and loganin) and a number of polyphenols, including cornusiin A, B, and C, and monomeric and trimeric hydrolysable tannins [2, 3]. Morroniside and loganin extracted from *C. officinalis *has been proposed to prevent oxidative stress [4].* C. officinalis, *or its constituents, is thought to enhance liver and kidney function and has been reported to exhibit antihypertriglyceridemic activity [5], and antioxidation effects [4, 6].

Acetaminophen (APAP) is widely prescribed as an analgesic and antipyretic drug in the clinic and is sold in numerous over-the-counter preparations as a single compound or in combination with other medications [7, 8]. At normal doses, APAP is metabolized by cytochrome P450 (CYP) to form the highly reactive species, *N*-acetyl-*p*-benzoquinone imine (NAPQI), which under normal conditions is readily detoxified by conjugation with glutathione (GSH). However, in humans and mice, high doses of APAP saturate detoxification pathways, leading to hepatic glutathione depletion and excessive production of NAPQI, which freely binds to cellular molecules [9]. 

Reactive oxygen species (ROS) are causally related to oxidative stress. Many studies have demonstrated that overproduction of ROS can further aggravate oxidative stress and have implicated ROS in a number disease processes, including heart disease, diabetes, liver injury, cancer, and aging [10–14]. Maintaining the balance between ROS and antioxidant enzymes, such as superoxide dismutase (SOD), catalase (CAT), and glutathione peroxidase (GPx), is, therefore, crucial and could be an important mechanism for preventing damage by oxidative stress. This balance has been suggested to have an important role in preventing APAP toxicity [15].

In recent years, an increasing attention has been focused on an “emerging molecule”: heme oxygenase-1 (HO-1), the rate limiting enzyme in the breakdown of heme into carbon monoxide, iron and bilirubin [16]. Ho-1 has been shown to be induced during oxidative injury, and its induction acts as an important cellular defense mechanism against such injuries [17]. It is noteworthy that induction of HO-1 expression contributes to protection against liver damage induced by several chemical compounds such as acetaminophen, carbon tetrachloride and heavy metals, suggesting HO-1 induction as a prominent cellular endeavor for hepatoprotection [18]. However, the physiological effects of *C. officinalis *in the context of chemically induced liver injury and possible involvement of HO-1 have not been determined. 

Therefore, we aimed to evaluate the protective effects of *C. officinalis *on APAP-induced hepatotoxicity in mice with emphasis on HO-1 induction, which may help to determine an optimal condition for further research.

## 2. Materials and Methods

### 2.1. Plant Material

The fruit of* C. officinalis *was purchased from Icheon, Korea, authenticated by Prof. Young Bae Seo (Daejeon Univ) and stored as a voucher specimen in the Herbarium of herbal medicine EBM research center, South Korea, with the issue no., KIOM2008CF002.

### 2.2. Chemicals and Reagents

Morroniside and loganin were purchased from NPC BioTechnology (Yeongi, Korea) and Wako (Osaka, Japan), respectively. The purity of each compound was determined to be above 98% by HPLC analysis. HPLC-grade reagents, methanol, acetonitrile, and water were obtained from J.T. Baker (Phillipsburg, NJ, USA). Other chemicals were of analytical grade.

### 2.3. Sample Preparation

*C. officinalis *was cut into small pieces and extracted twice with 70% ethanol at room temperature. The solution was filtered through filter paper and evaporated *in vacuo* to yield a powered extract (yield = 44.0%). The lyophilized powders of ethanolic extracts of *C. officinalis *(ECO) were dissolved in distilled water before oral administration to experimental animals.

For HPLC analysis, the plant material was powdered and sieved through a 600 *μ*m sieve (no. 30) prior to analysis. The sample powder was weighed (1.0 g) into a 100 mL flask and 100% methanol was added to the volumetric mark. The mixture was sonicated for 60 min at room temperature. After extraction, the mixture was filtered through a 0.2 *μ*m membrane filter. The resulting solution was used HPLC analysis.

### 2.4. Chromatographic System

Analysis was performed using a Shimadzu LC-20A HPLC system (Shimadzu Co., Kyoto, Japan), consisting of a solvent delivery unit, an online degasser, a column oven, an autosampler, and a PDA detector. The data processor employed LCsolution software (Version 1.24). The analytical column used was a Gemini C18 (250 × 4.6 mm; particle size 5 *μ*m, Phenomenex, Torrance, Calif, USA). The mobile phases consisted of solvent A (H_2_O) and solvent B (Acetonitrile) with the isocratic elution. The column temperature was maintained at 40°C. The analysis was carried out at a flowrate of 1.0 mL/min with PDA detection from 190–400 nm. The injection volume was 10 *μ*L.

### 2.5. Standard Solution and Calibration Curves

Standard stock solutions of morroniside and loganin (all 1,000 *μ*g/mL) were prepared in methanol and held stored 4°C. Working standard solutions were prepared by serial dilution of stock solutions with methanol. All calibration curves were obtained from assessment of peak areas of standard solutions in the concentration ranges: morroniside, 2.34–300.00 *μ*g/mL; loganin, 2.34–300.00 *μ*g/mL.

### 2.6. Animal Experimental Design

Five-week-old pathogen-free male BALB/c mice, routinely screened serologically for relevant pathogens, were purchased from the Orient Bio Co. (Seongnam, Korea). Mice were maintained in an animal facility under standard laboratory conditions for 1 week prior to experiments and were provided with water and standard chow *ad libitum*. Prior to treatment with APAP, mice were fasted, as indicated below. All experimental procedures were carried out in accordance with NIH Guidelines for the Care and Use of Laboratory Animals, and all animal handling conformed to the requirements of the National Animal Welfare Law of Korea.

Mice were divided into the following treatment groups (*n* = 8 or 9 mice/group): (1) control group, (2) APAP group, (3) silymarin + APAP group, (4) ECO 100 + APAP group, (5) ECO 250 + APAP group, and (6) ECO 500 + APAP group, (7) ECO 500 group.

The mice in control and APAP groups were pretreated only with distilled water; the other five groups were pretreated daily with ECO (100, 250, or 500 mg/kg) or silymarin (200 mg/kg) orally for 7 consecutive days. Silymarin has been used in clinical practice for the treatment of toxic liver disease [19]; thus, in this study, it was used as a positive control. Six hours after the final treatment, acute liver injury was induced in all groups, except the ECO500 group, by intraperitoneal (i.p) injection of 200 mg/kg APAP (Sigma-Aldrich Co., Mo, USA) in warm phosphate-buffered saline (PBS, pH 7.4). The mice were starved for 18 hr after APAP treatment and then were sacrificed. Blood was collected from abdominal veins, and plasma was separated and stored at −80°C for later analysis of biochemical parameters. The liver was quickly excised and divided into portions. Liver pieces were stored at −80°C for analysis of enzymes or fixed in 10% neutralized formalin for histological examination. 

### 2.7. Preparation of Liver Tissue Homogenate and Determination of Protein Content

Liver tissue (100 mg) was added to 500 *μ*L of 50 mM Tris buffer (pH 7.4) containing 1 mM EDTA and 10 *μ*g/mL leupeptin and was homogenized using an ultrasonic homogenizer (Sonoplus, Bandelin, Berlin, Germany). The homogenate was centrifuged at 10,000 x g for 15 min at 4°C, and the upper layer was collected and stored at −80°C for SOD, CAT, HO-1, and GSH assays. The total protein concentration in collected supernatant was determined colorimetrically using a Bio-Rad DC protein assay kit (Bio-Rad Laboratories, Calif, USA) to allow antioxidant enzymatic activity to be expressed on a per-gram-protein basis. 

### 2.8. Biochemical Analysis of Plasma Marker Enzymes

Eighteen hours after APAP treatment, plasma was collected and separated for measurement of aspartate transaminase (AST), alanine transaminase (ALT), and lactate dehydrogenase (LDH) enzymatic activities. Plasma was collected following centrifugation of blood for 10 min at 10,000 rpm. The levels of AST, ALT, and LDH were determined using an auto-analyzer (Beckman, Calif, USA, or ADVIA, Tokyo, Japan).

### 2.9. Lipid Peroxidation in Liver

Lipid peroxidation was analyzed by the formation of malondialdehyde (MDA) using a commercial kit (Cayman Chemical Company, Mich, USA) according to the manufacturer's protocol. Briefly, liver tissue was homogenized in RIPA buffer (50 mM Tris-Cl, pH 8.0, 150 mM NaCl, 1% NP-40, 5 mM EDTA, 1 mM PMSF) and centrifuged at 1,600 × g for 10 min at 4°C. Supernatant (0.1 mL) was mixed with a sodium dodecyl sulfate solution (0.1 mL) and color reagent (4 mL). The mixture was heated to 100°C for 1 hr and then rapidly cooled. Absorbance was measured at 532 nm using a microtiter plate reader (Bio-Rad Laboratories, UK).

### 2.10. Determination of SOD, CAT, and GSH in Liver

Liver tissue supernatants obtained from the liver tissue homogenization were used to determine the SOD, CAT, and HO-1 activities. The SOD activity in the liver tissues was determined using a SOD assay kit (Cayman Chemical Co., Mich, USA) according to the manufacturer's protocol. CAT activity in liver tissue was determined with a Catalase assay kit (Cayman Chemical Co., Mich, USA) according to the manufacturer's instruction. GSH level of liver tissues was measured using liver tissue supernatant with a commercial kit (Cayman Chemical Co., Mich, USA) according to the manufacturer's instructions. The absorbance was measured at 405 nm using a microtiter plate reader.

### 2.11. Measurement of HO-1 Activity

HO-1 activity in the liver tissues was measured using a Mouse Heme Oxygenase-1 EIA Kit (Takara Bio, Shiga, Japan) according to manufacturer's protocol. 

### 2.12. Liver Index and Histopathological Analysis

The liver index was calculated as liver weight divided by body weight. Liver tissues fixed in formalin were embedded in paraffin, cut into 4-*μ*m thick sections, mounted on glass slides, and stained with hematoxylin and eosin solution (H & E) (Sigma-Aldrich Co., Mo, USA). Stained sections were coverslipped using Dako-mounting medium (Dakocytomation, Calif, USA).

### 2.13. Statistical Analysis

All results are presented as mean ± SEM. Statistical analyses of the data were carried out using ANOVA and Bonferroni multiple comparison analysis, and values of *P*  <  0.05 were considered statistically significant.

## 3. Results

### 3.1. Optimization of the Chromatographic Conditions

**Figure 1:**
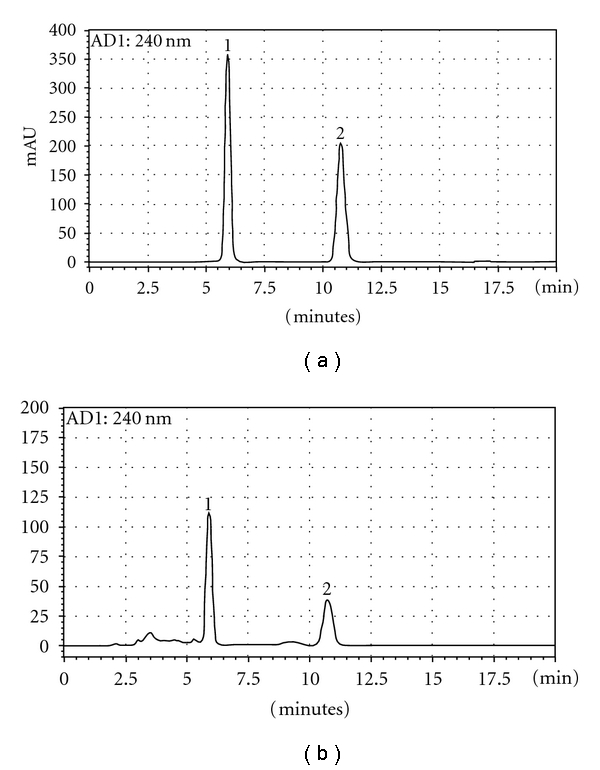
HPLC chromatogram of standard mixture (a) and *C. officinalis* (b) at 240 nm. Morroniside (1), loganin (2). *C. officinalis *and two standards were subjected to HPLC analysis. A Gemini C18 (250 × 4.6 mm) column was eluted with solvents A (H_2_O) and B (Acetonitrile) at flow rate of 1.0 mL/min.

We obtained satisfactory separation using mobile phases consisting of solvent A (H_2_O) and solvent B (Acetonitrile). The HPLC chromatogram of reference compounds and *C. officinalis* extract was shown in Figure 1. Using optimized chromatography conditions, two compounds were eluted before 20 min and showed good resolution in sample analysis.

### 3.2. HPLC Determination of Morroniside and Loganin from *C. officinalis*

**Figure 2:**
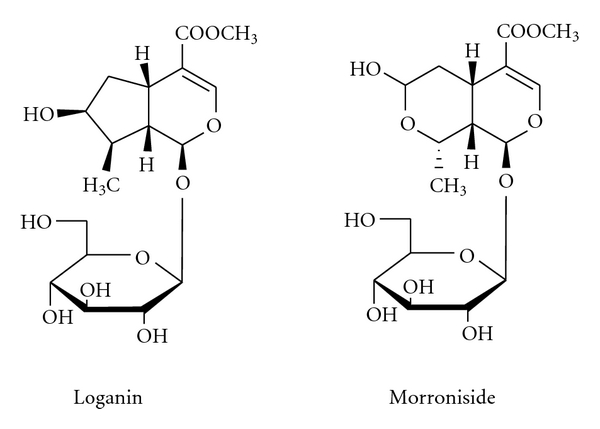
Chemical structures of major constituents in *C. officinalis*.

The standard curves for morroniside and loganin are *Y* = 19978.75*x* + 22514.05  (*R* ^2^ = 1.0000) and *Y* = 17047.30*x* + 21728.07  (*R* ^2^ = 0.9999), respectively. Retention time of morroniside and loganin were 5.908 min and 10.739 min, respectively. Chemical structures of major constituents in *C. officinalis* are shown in Figure 2. Figure 1 shows chromatograms of reference compounds and *C. officinalis* extract, with detection of eluent at 240 nm. The contents (*n* = 3) of morroniside and loganin were 9.63 mg/g and 5.82 mg/g, respectively, and summarized in Table 1. 

### 3.3. Biochemical Analysis of Plasma Marker Enzymes

**Figure 3:**
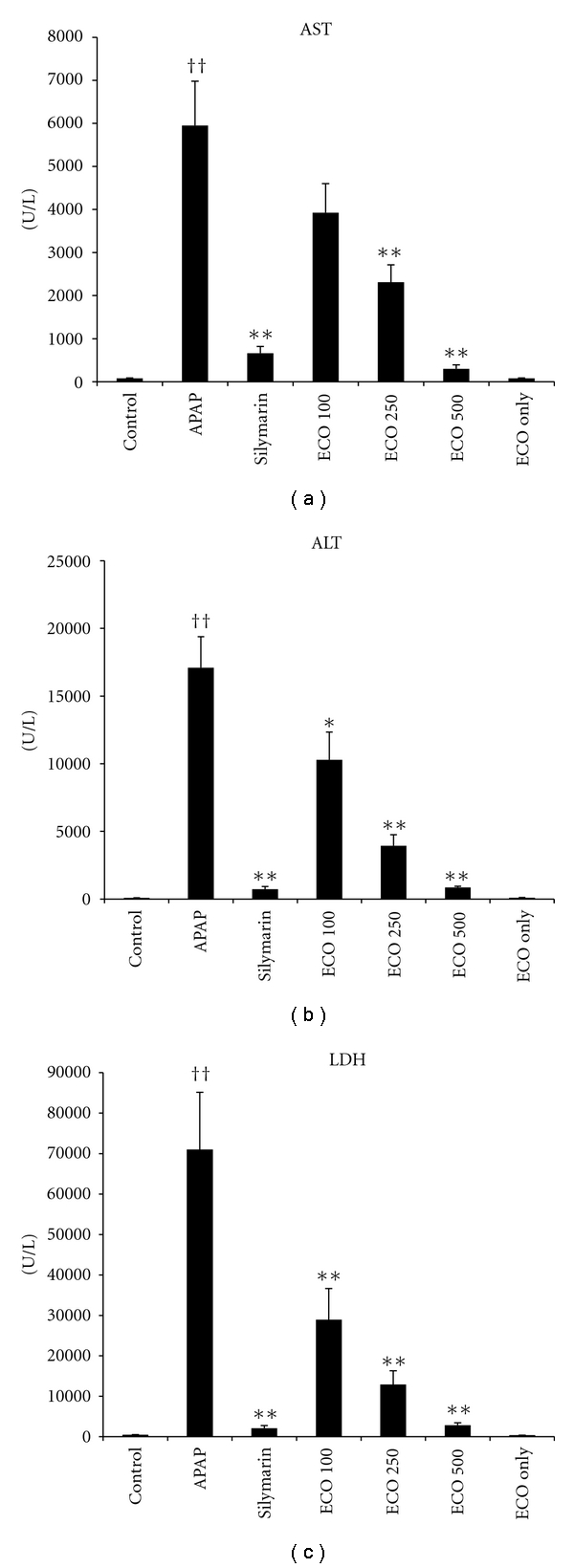
Protective effect of ECO on APAP-induced hepatotoxicity: plasma enzymes. Control, D.W treated/PBS injected; APAP, D.W treated/APAP 200 mg/kg injected; Silymarin, silymarin 200 mg/kg treated/APAP 200 mg/kg injected; ECO 100, ECO 100 mg/kg treated/APAP 200 mg/kg injected, ECO 250, ECO 250 mg/kg treated/APAP 200 mg/kg injected, ECO 500, ECO 500 mg/kg treated/APAP 200 mg/kg injected, ECO only, ECO 500 mg treated/PBS injected. AST, aspartate aminotransferase; ALT, alanine aminotransferase; LDH, lactate dehydrogenase. Values are expressed as means ± SEM (*n* = 8 or 9 mice/group). *Significant difference from Control, *P *< 0.05; ^†^significant difference from APAP, *P *< 0.05.

Plasma levels of AST, ALT, and LDH were determined as measures of liver function. A single administration of APAP induced severe hepatic injury, as shown by marked increases in AST, ALT, and LDH levels (*P*  <  0.01). Pretreatment with ECO significantly reduced plasma levels of AST, ALT, and LDH in a dose-dependent manner (*P*  <  0.05 or 0.01). The recovery from APAP-induced toxicity produced by treatment with ECO was similar to that seen with silymarin treatment (Figure 3).

### 3.4. Lipid Peroxidation in Liver

**Figure 4:**
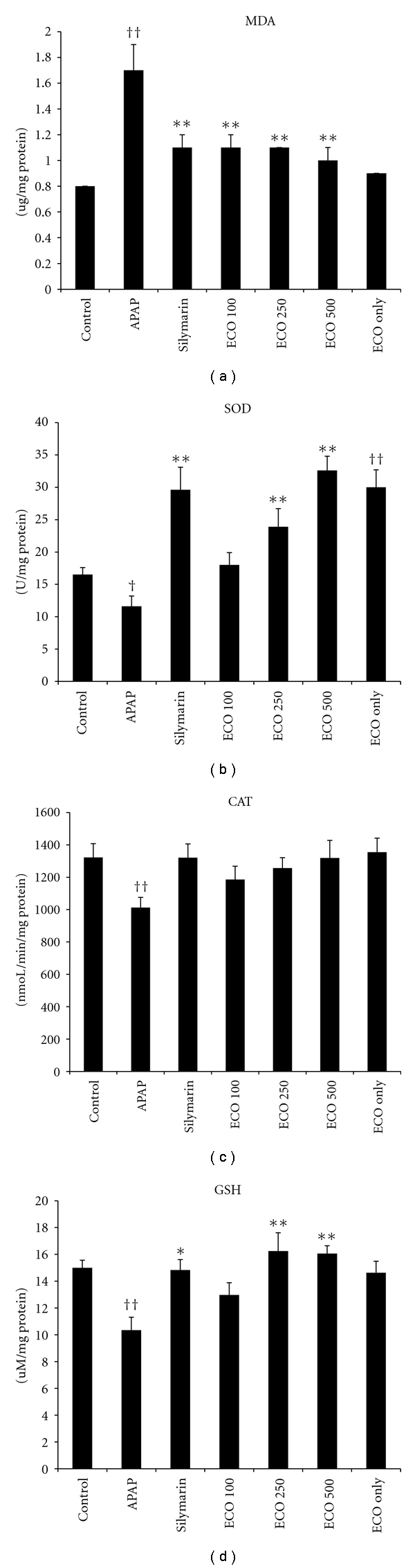
Protective effect of ECO on APAP-induced hepatotoxicity: lipid peroxidation and antioxidative exzyme activities. Control, D.W treated/PBS injected; APAP, D.W treated/APAP 200 mg/kg injected; Silymarin, silymarin 200 mg/kg treated/APAP 200 mg/kg injected; ECO 100, ECO 100 mg/kg treated/APAP 200 mg/kg injected, ECO 250, ECO 250 mg/kg treated/APAP 200 mg/kg injected, ECO 500, ECO 500 mg/kg treated/APAP 200 mg/kg injected, ECO only, ECO 500 mg treated/PBS injected. Values are expressed as means ± SEM (*n* = 8 or 9 mice/group). *Significant difference from Control, *P*  <  0.05; ^†^significant difference from APAP, *P*  <  0.05.

The lipid peroxidation was examined by determining malondialdehyde (MDA) content in liver tissue homogenate. The MDA content in APAP group was significantly higher than that in the control group (approximately 206% of control). In the ECO 100, ECO 250, ECO 500, and silymarin groups, MDA decreased by 37.5, 36.5, 41.4, and 37.3%, respectively, compared to APAP group (Figure 4(a)).

### 3.5. Determination of SOD, CAT, and GSH in Liver

We determined the level of SOD, CAT, and GSH in liver tissues. ECO treatment significantly prevented inhibition of SOD activity caused by APAP toxicity compared to the APAP group (*P*  <  0.01) (Figure 4(b)). We found sustained level of GSH components in the ECO-treated groups compared to the APAP group (*P*  <  0.01) (Figure 4(d)). 

### 3.6. Measurement of HO-1 Activity

HO-1 is an important component of the defense against oxidative stress. We therefore examined the effect of ECO on HO-1 activity. HO-1 activities increased markedly in the livers of mice in ECO 500 only group. 

**Figure 5:**
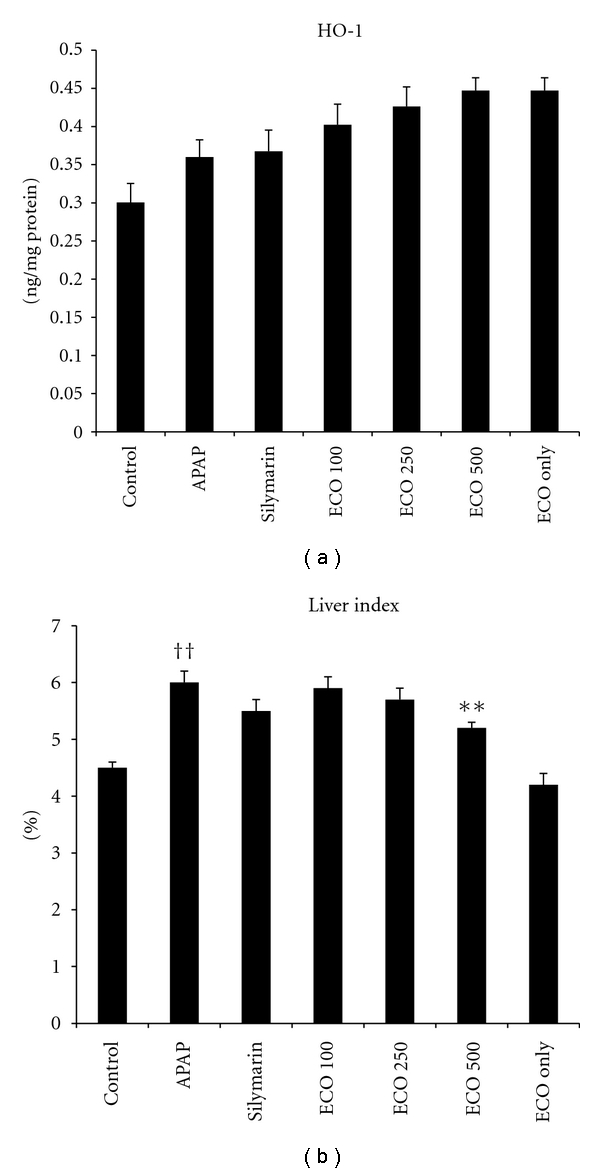
Protective effect of ECO on APAP-induced hepatotoxicity: HO-1 and liver index. Control, D.W treated/PBS injected; APAP, D.W treated/APAP 200 mg/kg injected; Silymarin, silymarin 200 mg/kg treated/APAP 200 mg/kg injected; ECO 100, ECO 100 mg/kg treated/APAP 200 mg/kg injected, ECO 250, ECO 250 mg/kg treated/APAP 200 mg/kg injected, ECO 500, ECO 500 mg/kg treated/APAP 200 mg/kg injected, ECO only, ECO 500 mg treated/PBS injected. Values are expressed as means ± SEM (*n* = 8 or 9 mice/group). *Significant difference from Control, *P*  <  0.05; ^†^significant difference from APAP, *P*  <  0.05.

We next investigated the effect of ECO on HO-1 activities in APAP-treated animals. Interestingly, the hepatic HO-1 activities in APAP group were higher than those of control group. Even though it was not significant, ECO 100, ECO 250, and ECO 500 group also resulted in further elevation of the APAP-induced HO-1 activities (Figure 5(a)). These results demonstrate that ECO treatment further augments HO-1 activities induced by APAP treatment and suggest that this augmented HO-1 activities may be important for the ECO-based hepatoprotection.

### 3.7. Liver Index and Histopathological Analysis

**Figure 6:**
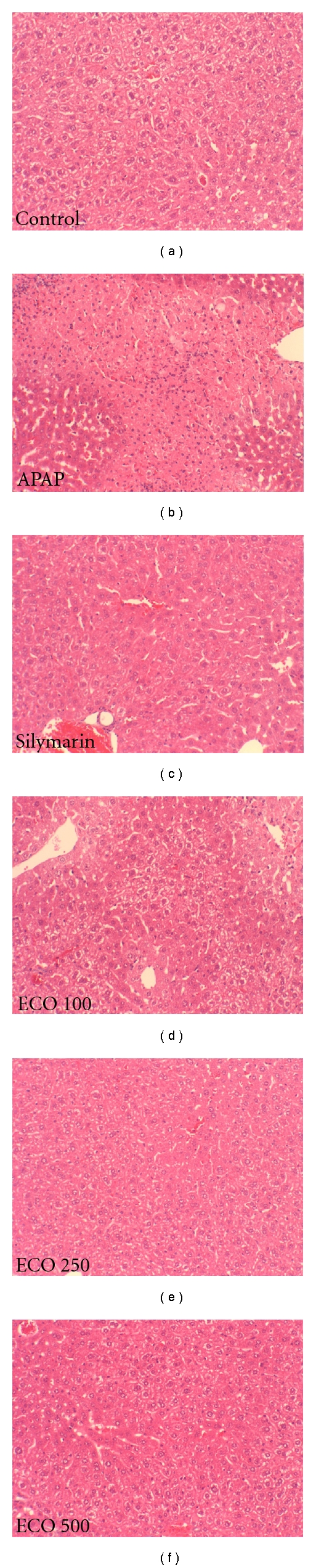
Protective effect of ECO on APAP-induced hepatotoxicity: H & E staining, magnification, ×200. Control, D.W treated/PBS injected; APAP, D.W treated/APAP 200 mg/kg injected; Silymarin, silymarin 200 mg/kg treated/APAP 200 mg/kg injected; ECO 100, ECO 100 mg/kg treated/APAP 200 mg/kg injected, ECO 250, ECO 250 mg/kg treated/APAP 200 mg/kg injected, ECO 500, ECO 500 mg/kg treated/APAP 200 mg/kg injected, ECO only, ECO 500 mg treated/PBS injected.

The liver index increased significantly in the APAP group (*P*  <  0.01), but this effect was significantly ameliorated by pretreatment with of ECO at 500 mg/kg (*P*  <  0.01) (Figure 5(b)). Liver sections stained with H & E showed features typical of inflammatory hepatic tissues, including centrilobular necrosis, confirming the hepatic damage indicated by biochemical and enzymatic assays (Figure 6). In the three ECO treatment groups, hepatocyte degeneration, necrosis, and infiltration of inflammatory cells were all reduced in a dose-dependent manner, similar to results in tissues from silymarin-treated mice. 

## 4. Discussion

*C. officinalis *or its constituents have been reported to possess potent antioxidant properties, including hydroxyl radical scavenging activity [4, 6]. However, potential hepatoprotective effects and the possible involvement of HO-1 have not been investigated. The present study showed for the first time that ECO has hepatoprotective properties, as evidenced by the significant inhibition of APAP-induced changes in liver biochemical parameters, antioxidant enzymatic activities, and lipid peroxidation product. We further show that HO-1 induction may be at least partly responsible for the action of ECO. We analyzed two compounds of *C. officinalis* such as morroniside and loganin using HPLC. Our result showed good separation in *C. officinalis *. On the other hand, we must perform activity tests for these major compounds.

APAP is a well-known antipyretic and analgesic agent, which is safe in therapeutic doses but can produce fatal hepatic necrosis in experimental animals and humans [20, 21] and is employed as an experimental hepatotoxic agent. 

Oxidative stress caused by APAP results in the release of LDH, a marker of cell damage, and the release of several soluble products, including ALT, and AST [20]. The estimation of enzymes in the serum is a useful quantitative marker of the extent and type of hepatocellular damage. The mice treated with an overdose of APAP developed significant hepatic damage, which was observed by a substantial increase in the concentration of serum enzymes (AST, ALT, and LDH). Administration of ECO after APAP treatment resulted in a significant reduction (*P  *<  0.05 or *P  *<  0.01) of APAP-induced elevation of AST, ALT and LDH and appears to be protective in reducing the injurious effect of APAP.

The MDA is a good indicator of the degree of lipid peroxidation [22], which is closely related to APAP-induced tissue damage. In the present study, we also observed significant increase (*P  *<  0.05) in the levels of MDA in liver, which was decreased by the administration of ECO. This might due to hydroxyl radicals scavenging activities of ECO. 

GSH is the major nonenzymatic antioxidant and regulator of intracellular redox homeostasis, ubiquitously present in all cell types [23]. The depletion of cellular GSH in the liver cells is known to play an important role in APAP toxicity [24]. APAP administration leads to a significant decrease in the glutathione level which can be an important factor in the APAP toxicity. The mechanism of hepatoprotection by ECO against APAP toxicity might be due to restoration of the GSH level. 

SOD catalyses the dismutation of superoxide anion to H_2_O_2_ and O_2_. Because H_2_O_2_ is still harmful to cells, CAT and GPx further catalyse the decomposition of H_2_O_2_ to water [25]. Thus, the coordinate actions of various cellular antioxidants in mammalian cells are critical for effectively detoxifying free radicals. APAP administration to mice declined antioxidant capacity of the mice liver as evinced in decreased activity of the antioxidant enzymes. ECO pretreatment prevented the reduction in the antioxidant enzyme activities and consequent oxidative damage to the liver.

HO-1 has been shown to be protective in several disparate models of hepatic injuries. Some HO-1 inducers have potential for use as effective preconditioning agents in solid organ transplantation. Curcumin, a candidate pharmacological preconditioning agent, was recently demonstrated to protect hepatocytes against oxidative injury and this protection was mediated through HO-1 induction [26]. It has been reported that expression of HO-1 is rapidly upregulated in the liver following administration of toxic doses of acetaminophen [27, 28]. Chiu et al. demonstrated a time dependent induction of HO-1 in the liver following treatment of rats with a toxic dose of acetaminophen, and pretreatment with hemin prevented acetaminophen hepatotoxicity [29]. In this study, pretreatment of ECO resulted in further elevation of the APAP-induced HO-1 activities but it was not significant. 

In addition, pretreatment with ECO prevented hepatic necrosis, an important histopathological feature of APAP-induced hepatotoxicity [30].

The present study has demonstrated that the ECO exerts a hepatoprotective effect against APAP-induced hepatotoxicity in mice. Increased levels of antioxidant enzymes and a reduction in the amount of lipid peroxides are likely to be the major mechanisms by which ECO prevents development of the liver damage induced by APAP.

## Figures and Tables

**Table 1:** Contents of identified compounds of *C. officinalis* by HPLC (*n* = 3).

Compound	RT (min)	Content (mg/g)	SD	RSD (%)
Morroniside	5.902	9.63	0.09	0.95
Loganin	10.728	5.82	0.04	0.68
